# Clinical and laboratory characteristics of asymptomatic and symptomatic neurosyphilis in HIV-infected patients: A retrospective study in China

**DOI:** 10.1097/MD.0000000000039617

**Published:** 2024-09-06

**Authors:** Ran Miao, Wenjing Zhang, Xinghuan Ding, Wurong Li, Lei Zhang, Cheng Kou, Ning Han, Yuming Huang

**Affiliations:** a Department of Neurology, Beijing Ditan Hospital, Capital Medical University, Beijing, China; b National Center for Infectious Diseases, Beijing Ditan Hospital, Capital Medical University, Beijing, China; c Department of Neurosurgery, Beijing Ditan Hospital, Capital Medical University, Beijing, China; d Department of Infectious Diseases, Beijing Ditan Hospital, Capital Medical University, Beijing, China.

**Keywords:** asymptomatic neurosyphilis, human immunodeficiency virus, symptomatic neurosyphilis

## Abstract

There are high rates of human immunodeficiency virus (HIV) and *Treponema pallidum* coinfection, HIV can increase the incidence and disability rate of neurosyphilis. However, there is a lack of data about the risk factors associated with the development of symptomatic neurosyphilis (SNS). We retrospectively reviewed the medical records of inpatients with concurrent syphilis and HIV infection who underwent a lumbar puncture and completed cerebrospinal fluid (CSF) examination. Sixty inpatients were consecutively enrolled from Beijing Ditan Hospital between January 2015 and March 2023. The clinical and laboratory features were evaluated between the SNS and asymptomatic neurosyphilis (ANS) groups. All patients were male, 25% (15/60) patients were diagnosed with ANS, and 75% (45/60) patients were diagnosed with SNS. Meningovascular neurosyphilis was the most prevalent clinical form in this study. Age, CD4 cell count, highly active antiretroviral therapy use, and serum HIV viral load showed no statistically significant differences between the 2 groups. The SNS group lacked early detection of syphilis (*P* < .001) and did not get previous adequate therapy for syphilis (*P* < .001) than the ANS group, as well as a higher initial serum toluidine red unheated serum test (TRUST) titer, current serum TRUST titer, CSF white blood cell count (WBC), protein concentration, and CSF TRUST titer (*P* = .014, *P* = .042, *P* = .01, *P* = .007, and *P* = .007, respectively). In multivariable logistic regression, high CSF WBC count (odds ratio = 1.08; *P* = .032) and previous treatment of syphilis (odds ratio = 0.01; *P* = .049) related to the SNS. Lack of antisyphilis treatment in the early stage of syphilis and a higher CSF WBC count are related risk factors for SNS in HIV-infected patients. Meningovascular neurosyphilis should get more attention in young patients with cryptogenic stroke.

## 1. Introduction

Neurosyphilis is caused by *Treponema pallidum* invades the central nervous system (CNS) and can occur at any time after infection. NS includes asymptomatic neurosyphilis (ANS), symptomatic syphilitic meningitis, meningovascular neurosyphilis, ocular syphilis, otologic syphilis, intracranial gummas, general paresis, and tabes dorsalis.^[1]^ Neurosyphilis is of particular concern in human immunodeficiency virus (HIV) infected individuals for several reasons, including (a) HIV and syphilis are transmitted in the same way, which leads to high rates of HIV and *Treponema pallidum* coinfection; (b) the difficulties in diagnosing neurosyphilis in these patients; (c) the potential increased risk of failing therapy for early syphilis, which may result in the development of early neurosyphilis; and (d) the possibly higher rates of treatment failure. All of these factors may contribute to long-term morbidity in patients with HIV infection. In China, there were 1.053 million people living with HIV, syphilis is the most common sexually transmitted infection among patients with HIV in China.^[2]^ Compared with without HIV infection, neurosyphilis was estimated to occur twice in patients with coinfection.^[3]^ From 2007 to 2017, the morbidity of syphilis showed a significant upward trend, from 15.8834/100,000 to 34.4867/100,000 in China.^[4]^

However, the relationship between ANS and symptomatic neurosyphilis (SNS) in patients with HIV is not well understood. We conducted this retrospective study to analyze the differences between ANS and SNS to better understand the clinical characteristics and laboratory of neurosyphilis in patients with HIV, and investigate the risk factors of SNS.

## 2. Methods

### 2.1. Study design

We retrospectively analyzed data from inpatient clinical records at the Department of Neurology, Beijing Ditan Hospital, Capital Medical University, and reviewed the neurosyphilis of all HIV-infected patients between January 2015 and March 2023. To rule out neurosyphilis, all patients underwent lumbar puncture, either because they had neurological or ophthalmic symptoms or signs or because they had a serofast status without any clinical symptoms or signs. Clinical and laboratory data were collected, including age, sex, clinical manifestations, comorbidity, HIV infection risks, highly active antiretroviral therapy (HAART) use, and laboratory and imaging materials. To assess the effect of HAART, the HAART-positive group included patients who received HAART therapy for 6 months or more before the diagnosis of neurosyphilis. HIV infection was diagnosed using an anti-HIV antibody test, which was screened using enzyme-linked immunosorbent assay and confirmed using western blotting. The most commonly used nontreponemal test to diagnose neurosyphilis from cerebrospinal fluid (CSF) is the venereal disease research laboratory (VDRL) test. However, it has low sensitivity, and the procedure is complicated and time-consuming. Additionally, the Chinese Food and Drug Administration has not approved any commercial VDRL reagent for use in CSF analysis. Gu et al suggested that toluidine red unheated serum test (TRUST) could be used as an alternative test for neurosyphilis diagnosis when VDRL is unavailable (7). All patients were treated with benzyl penicillin 24 million units intravenous injection daily, as 4 million units every 4 hours during 14 days. After detailed evaluation based on the diagnostic criteria, a total of sixty subjects were included in this study. This study was approved by the ethics committee of Beijing Ditan Hospital.

### 2.2. Diagnostic criteria

Neurosyphilis was categorized as ANS or SNS according to the absence or presence of neurological or ophthalmic symptoms or signs. The diagnostic criteria for neurosyphilis in patients with HIV were based on CDC guidelines in the USA and Europe.^[5,6]^ Both serum *T pallidum* particle agglutination (TPPA) and TRUST were reactive; syphilis at any stage with reactive TRUST in the CSF was confirmed as neurosyphilis. If CSF TRUST results were negative, both of the following abnormal findings were necessary: (a) positive CSF TPPA, and (b) CSF white blood cell (WBC) > 20 cells/µL or protein > 50 mg/dL in the absence of other known causes of these abnormalities. Patients were excluded if their clinical manifestations were not caused by neurosyphilis or had concurrent CNS infections. Eye findings were reviewed by formal ophthalmological examination, and ocular syphilis was diagnosed by a specialized ophthalmologist.

### 2.3. Statistical analysis

IBM SPSS 22.0 version (SPSS Inc., Chicago, IL) was used to analyze the data. Continuous variables with a normal distribution were described using mean ± standard deviation (x ± SD) and compared using an independent two-sample *t* test. Continuous variables with a skewed distribution were presented using the median and interquartile range (IQR) and compared by the Mann–Whitney *U* test. Categorical variables were expressed as numbers (percentage), and compared using the chi-square (x^2^) test. All statistical assessments were two-tailed, and statistical significance was set at *P* < .05. The TRUST titer measured by the double dilution method was applied to analyze after a log transformation (log2 1/TRUST titer).

## 3. Results

**Figure 1.**
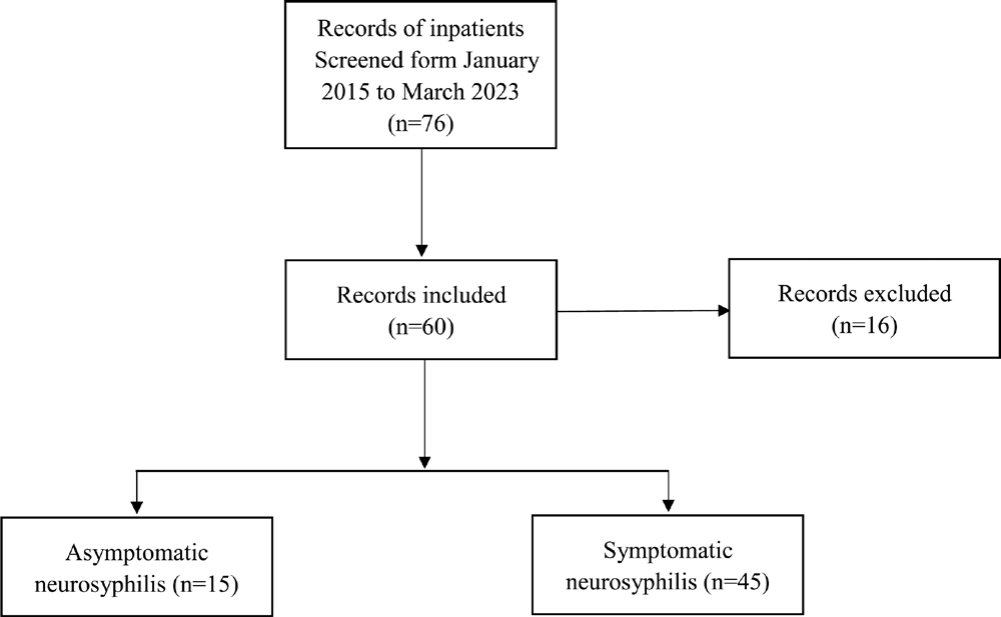
Flowchart of neurosyphilis patients in this study.

Sixteen patients were excluded in our study. The causes for exclusion were as follows: tuberculous meningitis (n = 2), tuberculous meningoencephalitis (n = 3), cryptococcal meningitis (n = 3), cytomegalovirus encephalitis (n = 4), progressive multifocal leukoencephalopathy (n = 2), and diffuse large B-cell lymphoma (n = 2) (Fig. 1).

Sixty patients were enrolled with positive TPPA in the serum and CSF. Fifteen (15%) patients were diagnosed with ANS, and the median age was 36 years old (IQR, 33–46 years). Of these patients, 9 patients had an early identification of syphilis and undergone benzathine penicillin G treatment prior to the diagnosis of neurosyphilis. The median CD4 cell count was 177 cells/µL (median and interquartile range [IOR], 107–360 cells/µL). Thirteen patients had available serum HIV viral load (median serum HIV viral load, 18308; IOR, 0–311,151) copies/mL. Seven patients had received HAART before neurosyphilis diagnosis. The median initial serum TRUST titer was 1:32 (IQR, 1:8–1:128) and the median current serum TRUST titer was 1:32 (IQR, 1:8–1:128) when a lumbar puncture was performed. Nine patients had positive CSF TRUST results, of which 5 had a TRUST titer of 1:1, 3 had a TRUST titer of 1:2, and 1 had a TRUST titer of 1:8. Five patients had a CSF WBC > 20/µL; the median CSF WBC count was 15 (IOR, 3–22)/µL. Seven patients had a CSF protein concentration >50 mg/dL; the median CSF protein concentration was 48.9 (IOR, 42.6–79.3) mg/dL (Table 1).

**Table 1:** Demographic and clinical information between the SNS and ANS groups.

Characteristic	SNS (45)	ANS (15)	*P* value
Age (years), median year (IOR)	36 (28.5, 47.5)	36 (33,46)	*P* = .334
Risks for HIV infection			
MSM(n, %)	9, 20%	6, 40%	*P* = .121
Heterosexual(n, %)	7, 15.6%	3, 20%	*P* = .689
IVDA(n, %)	0	0	
Unknown(n, %)	29, 64.4%	6, 40%	
Early identification of syphilis(n, %)	5, 11.1%	9, 60%	*P* < .001
Previous treatment of syphilis(n, %)	4, 8.9%	9, 60%	*P* < .001
HAART(n, %)	13, 28.9%	7, 46.7%	*P* = .206
CD4 cell count, median (IOR),/cells/µL	160 (66, 380)	177 (107, 360)	*P* = .489
CD4>200(n, %)	20, 44.4%	6, 40%	
200>CD4>50(n, %)	18, 40%	9, 60%	
CD4<50(n, %)	7, 15.6%	0, 0%	
Serum HIV virus load, median(IOR)/copies/mL	104,600 (1798, 257,374)	18,308 (0, 311,151)	*P* = .489
Initial serum TRUST titer, median (IOR)	1:128(1:32, 1:256)	1:32(1:8,1:128)	*P* = .014
Current serum TRUST titer, median (IOR)	1:128(1:32, 1:256)	1:32(1:8,1:128)	*P* = .042
CSF TRUST titer (positive: negative)	38：7	9：6	*P* = .047
CSF TRUST titer, median (IOR)	1:2(1:1, 1:4)	1:1(negative,1:2)	*P* = .007
CSF WBC count, median (IOR),/µL	35 (11.5, 72.5)	15 (3, 22)	*P* = .01
>20/µL (n, %)	28, 62.2%	5, 33.3%	*P* = .051
CSF protein concentration, median(IOR) mg/dL	90.5 (52.5, 110.6)	48.9 (42.6, 79.3)	*P* = .007
>50/mg/dL (n, %)	34, 75.6%	7, 46.7%	*P* = .037

ANS = asymptomatic neurosyphilis, CSF = cerebrospinal fluid, HAART = highly active antiretroviral therapy, IVDA = intravenous drug abuse, MSM = men who have sex with men, NA = not available, SNS = symptomatic neurosyphilis, TPPA = *Treponema pallidum* particle agglutination, TRUST = toluidine red unheated serum test, WBC = white blood cells.

Forty-five (75%) patients were diagnosed with SNS. Meningovascular syphilis (18 cases) was the most common type of SNS, followed by syphilitic meningitis (11 cases), ocular syphilis (8 cases), general paresis (5 cases), tabes dorsalis (2 cases), and intracranial syphilitic gumma (1 case). The most prevalent initial symptoms of SNS were limb weakness (13 cases), followed by headache (9 cases), vision impairment or loss (8 cases), impaired consciousness (3 cases), lighting pain in the legs and trunk (2 cases), personality change (2 cases), blepharoptosis (2 cases), cognitive impairment (2 cases), hearing impairment (1 case), and tremor (1 case). The most common signs were hemiplegia or hemiparesis (13 cases), followed by visual disorder (8 cases), sensory disturbance (7 cases), psychological and behavior disorders (6 cases), cognitive decline (5 cases), Argyll-Robertson pupil (4 cases), dysarthria (4 cases), dysaudia (2 cases), and tremor (1 case). The median age was 36 (IQR, 28.5–47.5) years old. Of these patients, 5 had an early identification of syphilis, 4 of them received benzathine penicillin G treatment prior to the diagnosis of neurosyphilis, and 41 had no treatment history. The median CD4 cell count was 160 cells/µL (IOR, 66–380 cells/µL). Forty-three patients had available serum HIV viral load (median serum HIV viral load, 104,600; IOR, 1798–257,374) copies/mL. Thirteen patients underwent HAART before neurosyphilis was diagnosed. The median initial serum TRUST titer was 1:128 (IQR, 1:32–1:256) and the median current serum TRUST titer was 1:128 (IQR, 1:32–1:256) when a lumbar puncture was performed. A total of 38 patients reported positive CSF TRUST, with TRUST values ranging from 1:1 to 1:2 to 1:4 to 1:8 to 1:16 in 10 patients, 7 patients, 12 patients, 7 patients, and 2 patients, respectively. Twenty-eight patients had CSF WBC count > 20/µL; the median CSF WBC count was 36 (IOR, 12.5–73.5)/µL. A CSF protein concentration >50 mg/dL was present in 34 cases; the median CSF protein concentration was 90.5 (IOR, 52.5–110.6) mg/dL (Table 1). As for ocular syphilis, 3 patients had anterior uveitis and 5 patients had posterior uveitis.

Thirty-four patients performed magnetic resonance imaging (MRI), and MRI findings of 6 patients were not used for further analysis because of comorbidities, including hypertension, hyperlipidemia and diabetes. Six patients had normal brain MRI findings, 22 patients presented with abnormal manifestations, including demyelination in 15 patients, infarct ischemic stroke in 14 patients, multiple cerebral infarctions in 4 patients, leukoaraiosis in 4 patients, cerebral atrophy in 6 patients, hydrocephalus in 4 cases, leptomeningeal enhancement in 2 patients, abnormal signals in spinal cord in 2 patients, subarachnoid hemorrhage in 1 patient and intracranial space occupying in 1 case.

The clinical features were evaluated between the SNS and ANS groups, it was found that the ANS group had more patients who had early identification of syphilis and received antisyphilis treatment than the SNS group (*P* < .001). Age, HIV infection risks, and HAART use did not differ significantly between the 2 groups (Table 1).

Regarding laboratory findings between the 2 groups, the SNS group’s initial and current serum TRUST titers were significantly higher than those of the ANS group (*P* = .014, and *P* = .042, respectively). In the CSF test, the SNS group had more positive CSF TRUST rate and substantially higher CSF WBC count, CSF protein concentration, and CSF TRUST titer than the ANS group (*P* = .047, *P* = .01, *P* = .007, and *P* = .007, respectively). There were no significant differences between the 2 groups in terms of CD4 cell count or serum HIV load (Table 1).

Bivariate analysis revealed that the following factors were more likely to be associated with the development of SNS: high initial serum TRUST titer (odds ratio [OR] = 1.01; *P* = .021), high CSF TRUST titer (OR = 1.46; *P* = .040), high CSF protein concentration (OR = 1.03; *P* = .012), and high CSF WBC count (OR = 1.03; *P* = .049). Previous treatment of syphilis reduce the likelihood of developing SNS (OR = 0.09; *P* < .001). In multivariable logistic regression, SNS related to the following factors: high CSF WBC count (OR = 1.08; *P* = .032) and previous treatment of syphilis (OR = 0.01; *P* = .049) (Table 2).

**Table 2:** Univariate and multivariate analysis for predictors of SNS groups.

Factors	Univariate			Multivariate
	*P*	OR (95% CI)	*P*	OR (95% CI)
Initial serum TRUST titer	.021	1.01 (1.01–1.01)	.090	1.03 (1.00–1.06)
CD4 cell count, cells/µL	.923	1.00 (1.00–1.00)	.549	1.00 (0.99–1.01)
Serum HIV virus load, copies/mL	.425	1.00 (1.00–1.00)	.055	1.00 (1.00–1.00)
Current serum TRUST titer	.067	1.01 (1.00–1.01)	.383	0.99 (0.96–1.02)
CSF TRUST titer	.040	1.46 (1.02–2.10)	.151	2.18 (0.75 ~ 6.33)
CSF WBC count/µL	.049	1.03 (1.01–1.05)	.032	1.08 (1.01–1.11)
CSF protein concentration, mg/dL	.012	1.03 (1.01–1.05)	.631	1.01 (0.96–1.16)
Previous treatment of syphilis, yes vs no	<.001	0.09 (0.02–0.38)	.049	0.01 (0.00–0.98)
HAART	.211	0.46 (0.14–1.54)	.064	45.42 (0.80–2586.73)

CI = confidence interval, CSF = cerebrospinal fluid, HAART = highly active antiretroviral therapy, OR = Odds Ratio, TRUST = toluidine red unheated serum test, WBC = white blood cells.

## 4. Discussion

Previous studies had suggested that HIV infection was associated with more frequent recurrence of syphilis following treatment and greater rates of syphilis recurrence after therapy.^[7,8]^ HIV can cause meningeal lesions that make it easier for *T pallidum* to cross the blood–brain barrier. Conversely, syphilis affects HIV disease and its treatment. For example, primary syphilitic ulcers increased the risk of HIV infection.^[9,10]^ Syphilis infection could increase HIV replication and worsen CD4 lymphocyte loss.^[11]^ Neurosyphilis may amplify the HIV viral load in the CSF, which may lead to a higher frequency of neurocognitive impairment.^[12]^ Several previous studies concerned with risk factors of neurosyphilis in patients with HIV found that neurosyphilis was more prevalent in male, higher serum rapid plasma regain titers, lower CD4 cell count, not received HAART, and untreated with syphilis.^[13,14]^ However, the risk factors associated with the development of SNS are still unclear. This study found lack of antisyphilis treatment and higher CSF WBC counts may be associated with the development of SNS.

Old age was identified as a risk factor for SNS in some previous literature, which may be associated with a longer duration of *T pallidum* infection, but we found no age difference between the 2 groups.^[15,16]^ Based on guidelines, early neurologic clinical manifestations (i.e., cranial nerve dysfunction, meningitis, stroke, acute altered mental status, and auditory or ophthalmic abnormalities) are usually present within the first few months or years of infection. Late neurologic manifestations (i.e., tabes dorsalis and general paresis) occur 10 to 30 years after infection.^[5]^ Therefore, the difference may be due to the difference in the main type of symptomatic neurosyphilis.

All participants in our study were male. We assume that the reason for the gender differences may be that unhealthy sexual behavior is more common in men.

In theory, disease duration should also be a risk factor for SNS, but it is difficult for patients to provide an exact time of syphilis infection. Therefore, this study did not consider the disease duration. We speculated that longer disease duration contributed to the development of SNS.

A retrospective analysis of 101 patients with concurrent syphilis and HIV infection showed that ocular syphilis occurs in 52% of the patients.^[17]^ Anterior uveitis was the most commonly observed ocular manifestation of syphilis, but in patients with concurrent syphilis and HIV infection, especially when the CD4 cell count is <200/mm^3^, posterior uveitis was more common.^[17]^ In this study, posterior uveitis was more frequent, but only 1 patient with posterior uveitis had a CD4 cell count below 200/mm^3^.

Meningovascular neurosyphilis was the most common clinical type of neurosyphilis observed in the present study. Most patients with meningovascular neurosyphilis were young adults without typical risk factors for cerebrovascular disease, and most cerebral infarctions occurred in the middle cerebral artery (MCA) (Table 3). The pathological basis of meningovascular neurosyphilis is endarteritis of the medium and large arteries (Hebner arteritis) and endarteritis of the small arteries and arterioles (Nissl-Alzheimer), which leads to inflammation and fibrosis in the adventitia.^[18]^ Progressive stenosis can lead to thrombosis and infarction. The MCA was the most commonly involved artery, followed by the basilar artery.^[19]^ Our research is consistent with previous literature. According to 1 study, the misdiagnosis rate of neurosyphilitic ischemic stroke was 80.95%; all patients with meningovascular neurosyphilis in our study were misdiagnosed with “cerebral stroke” at other hospitals (18). Clinicians should always consider the differential diagnosis of meningovascular neurosyphilis in stroke, especially in young and male patients with cryptogenic stroke.

**Table 3:** Symptoms and imaging findings of patients with meningovascular syphilis.

No	Age	Ischemic stroke risk factors	Symptoms	Lesion on CT/MRI	Vascular findings
1	36	Cigarette smoking	Disorders of consciousness	Pons, left cerebellum	NA
2	29	No risk factors	Motor aphasia, right-sided weakness	Left frontal lobe, left temporal lobe, left insular lobe, and leptomeningeal enhancement	High-grade narrowing of MCA
3	22	No risk factors	Right-sided weakness, right facial droop, and dysarthria	Left basal ganglia, left centrum semiovale	High-grade narrowing of MCA
4	37	Cigarette smoking	Dysarthria, left-sided weakness	Right centrum semiovale, pons, and left thalamus	Marked narrowing and irregularity of the BA
5	33	No risk factors	Headache, unresponsive	Left thalamus, left frontal lobe	NA
6	32	Cigarette smoking	Dysarthria, left hemiparesis	Right frontal lobe, right temporal lobe, and right parietal lobe	No evidence of arterial narrowing
7	54	Diabetes mellitus, hyperlipidemia, alcohol drinking	Slurred speech, right-sided weakness, and right-sided numbness on the body	Left cerebral peduncle, right pons	Moderate narrowing of the BA, unstable plaque
8	36	No risk factors	Dysarthria, right-sided weakness, and right facial droop	Right cerebellum, left pons, and bilateral thalamus	NA
9	32	Cigarette smoking	Vertigo	Left cerebral peduncle, right pons	NA
10	28	No risk factors	Disorders of consciousness, right hemiparesis	Left frontal lobe, left temporal lobe, left parietal lobe, and left insular lobe,	MCA occlusion
11	40	Cigarette smoking, alcohol drinking	Vertigo, right-sided weakness	Medulla oblongata, left basal ganglia	NA
12	34	No risk factors	Left-sided weakness	Right basal ganglia, right lateral ventricle	Right distal MCA occlusion, moderate narrowing of the left MCA
13	44	No risk factors	Headache, seizure, left oculomotor nerve palsy, and right hemiparesis	Left Caudate nucleus, left putamen nucleus, and subarachnoid hemorrhage	Aneurysm in the left PComA
14	58	Diabetes mellitus	Dysarthria, right facial droop, and right-sided weakness	Left frontal lobe, left parietal lobe, and left occipital lobe	High-grade narrowing of PCA
15	42	Hypertension, diabetes mellitus	Dysarthria, left-sided weakness	pons	High-grade narrowing of left VA
16	53	Cigarette smoking, alcohol drinking	Vertigo, nausea, and vomiting	pons	NA
17	47	Diabetes mellitus	Right-sided weakness, vertigo, and coma	Right pontine brachium	Stenosis of the bilateral ICA
18	57	Cigarette smoking	Dysarthria	Left frontal lobe, left temporal lobe	Atherosclerosis of the bilateral ICA

BA = basilar artery, ICA = internal carotid artery, MCA = middle cerebral artery, NA = not available, PCA = posterior cerebral artery, PComA = posterior Communicating Artery, VA = vertebral artery.

The ANS misdiagnosis rate among the HIV population may be high owing to its characteristics of being a “great imitator.” Therefore, it is important to identify asymptomatic patients before symptom development. Several studies have attempted to demonstrate the criteria for the performance of lumbar puncture in HIV and syphilis coinfected patients without neurologic symptoms, including serum rapid plasma regain ≥ 1:32, CD4 cell counts ≤ 350 cells/ml, HIV viral load ≥ 10,000 copies/mL, serologic nonresponse to antisyphilis treatment or concomitant ocular syphilis.^[14,17,20,21]^ In this study, 9 patients underwent lumbar puncture because their serum TRUST titer decreased by <4-fold after adequate benzathine penicillin G treatment, unrecognized CNS infections may be the reason for treatment failure.

Benzathine penicillin G treatment is effective in killing *T pallidum*, blocking the natural process of syphilis, and decreasing the incidence of neurosyphilis. According to our study, more patients in the SNS group significantly lacked benzathine penicillin G treatment in their early stage of syphilis infection than those in the ANS group (91.1% vs 40%, *P* < .001). The Current serum TRUST titer was significantly higher in SNS group, which may be due to the lack of antisyphilis treatment. Multivariate analysis revealed that previous benzathine penicillin G treatment reduced the risk of developing SNS (OR = 0.01, *P* = .049), indicating the importance of early treatment of syphilis.

The SNS group’s CSF WBC counts were significantly higher than the ANS group’s, further analysis showed that CSF WBC count was an independent risk factor for SNS (OR = 1.05 *P* = .032), suggesting that inflammatory activity was more obvious in patients with SNS. CSF TRUST was also positively correlated with neurosyphilis activity, and more patients in the SNS group had positive CSF TRUST and higher CSF TRUST titer than those in the ANS group, indicating that patients with SNS had higher disease activity. The level of CSF protein concentration responds to the destruction of the blood–brain barrier, and patients with SNS have higher levels of CSF protein concentration, which may indicate more severe blood–brain barrier damage. There was no significant difference in the CD4 cell count, serum HIV virus load and HAART use between the ANS and SNS groups, suggesting that the immune status of HIV patients may not affect neurosyphilis manifestation.

This study had certain limitations. Because this was a retrospective study, the baseline characteristics of the included patients were difficult to control. Many patients did not obtain data such as the possible route of HIV transmission, and a large number of patients did not undergo CSF HIV viral load test or imaging examinations. The sample size of this study was small and included only inpatients, resulting in insufficient test efficiency for statistical analysis. Further multi-center studies are needed to address this issue.

## 5. Conclusion

In conclusion, clinical and laboratory findings in the ANS and SNS groups were quite different. Lack of antisyphilis treatment in the early stage of syphilis and a higher CSF WBC count are related risk factors for SNS in HIV-infected patients. Meningovascular neurosyphilis should be considered in young patients with cryptogenic stroke.

## Author contributions

**Data curation:** Cheng Kou.

**Project administration:** Xinghuan Ding, Lei Zhang.

**Resources:** Ning Han.

**Supervision:** Wurong Li.

**Writing – original draft:** Ran Miao.

**Writing – review & editing:** Wenjing Zhang, Yuming Huang.
